# ﻿Three new species of the genus *Kockovaella* (Cuniculitremaceae, Tremellales) from the phylloplane in China

**DOI:** 10.3897/mycokeys.110.133084

**Published:** 2024-11-20

**Authors:** Chun-Yue Chai, Zhi-Wen Xi, Qiu-Hong Niu, Feng-Li Hui

**Affiliations:** 1 School of Life Science, Nanyang Normal University, Nanyang 473061, China Nanyang Normal University Nanyang China; 2 Research Center of Henan Provincial Agricultural Biomass Resource Engineering and Technology, Nanyang Normal University, Nanyang 473061, China Nanyang Normal University Nanyang China

**Keywords:** *Basidiomycota*, phylogenetic analysis, plant leaves, taxonomy, Tremellomycetes

## Abstract

*Kockovaella*, in the family Cuniculitremaceae of the order *Tremellales*, is a globally distributed genus of blastoconidia-forming fungi. Currently, 23 species have been described and accepted as members of the genus. In this study, five yeast strains were isolated from plant leaf surfaces collected in the Fujian and Guizhou Provinces of China and identified through a combination of morphological and molecular methods. The related phenotypic features and molecular phylogenetic analyses based on ITS, LSU, and RPB1 sequences demonstrated that they were members of three novel *Kockovaella* species: *K.iteae***sp. nov.**, *K.quanzhouensis***sp. nov.**, and *K.sambucuse***sp. nov.** These species were described in detail and discussed relative to other species. This study demonstrated the novel geographical distribution as well as the high species diversity of *Kockovaella* in China and offered more data for further studies in fungal systematics and evolution.

## ﻿Introduction

*Kockovaella* (*Tremellales*, Cuniculitremaceae), a ballistoconidiogenous anamorphic yeast genus, was first proposed by Nakase et al. (1991) to accommodate two species, *K.imperatae* and *K.thailandica*. Nine other species were subsequently identified in this genus (Canete-Gibas et al. 1998; Takashima and Nakase 1998; Luong et al. 2000; Fungsin et al. 2002). Prior phylogenetic investigations of the small subunit (SSU) rRNA gene demonstrated that *Kockovaella* was closely related to *Fellomyces* (Nakase et al. 1993). However, the division of *Kockovaella* and *Fellomyces* species into two genera is questionable because further phylogenetic studies indicate a close relationship between the species, and the only difference is the capacity of *Kockovaella* to produce ballistospores (Lopandic et al. 2011; Takashima and Nakase 2011). Nakase et al. (1993) and Nakase (2000) noted that the production of ballistospores may be influenced by cultivation methods and vary from clone to clone. Vegetative reproduction does not reflect the phylogenetic relationships, and a novel approach to the systematics of ballistosporous yeasts should be established (Lopandic et al. 2005a). Liu et al. (2015a) employed seven genes to reconstruct the phylogeny of most described anamorphic and teleomorphic tremellomycetous yeasts. Based on multi-gene phylogenies, eight nonballistoconidium-forming species previously assigned to *Fellomyces* (Lopandic et al. 2011) were transferred to *Kockovaella* (Liu et al. 2015a, b). The latest additions to the genus were *K.libkindii* from the cavity of the bromeliad *Vrieseaminarum* in Brazil (Gomes et al. 2016), *K.haikouensis*, *K.ischaemi*, and *K.nitrophila* from the phylloplane in China (Li et al. 2020).

All species of the genus *Kockovaella* are asexual morphs, which are morphologically characterized by the production of blastoconidia on stalk-like conidiophores and budding cells. Some species may produce ballistoconidia and poorly developed pseudohyphae (Lopandic et al. 2011; Takashima and Nakase 2011; Liu et al. 2015b). Physiologically, members of the genus cannot undergo fermentation, possess Q-10 as a predominant ubiquinone, and assimilate diverse carbon sources, but not nitrate (Liu et al. 2015b). Species in this genus can be differentiated via phenotypic characteristics and phylogenetic analyses (Liu et al. 2015b; Li et al. 2020).

To date, 23 species have been accepted as members of the genus *Kockovaella*, with most reported in tropical and subtropical regions, especially in Asia (Lopandic et al. 2011; Takashima and Nakase 2011; Li et al. 2020). Previously, only four species, *K.chinensis*, *K.fuzhouensis*, *K.lichenicola*, and *K.sichuanensis*, were reported in Fujian and Sichuan Provinces of China (Yue 1982; Prillinger et al. 1997). Recently, Li et al. (2020) identified eight *Kockovaella* species in Hainan and Yunnan Provinces, including three new species. Despite these findings, the genus’s diversity remains incompletely understood. In this study, five basidiomycetous yeast strains were obtained from Fujian and Guizhou Provinces. Phenotypic characteristics and molecular phylogenetic analyses determined that these strains represent three undescribed species of *Kockovaella*. The aim of the present study was to identify and describe these new taxa using an integrative taxonomic approach.

## ﻿Materials and methods

### ﻿Sample collection and yeast isolation

Leaf samples were collected in the Fujian and Guizhou Provinces of China. Yeast strains were isolated from leaf surfaces using the improved ballistospore-fall method outlined by Nakase and Takashima (1993). Fresh leaves were cut into small pieces and affixed with a thin layer of petroleum jelly to the inner lid of a Petri dish containing yeast extract-malt extract (YM) agar (0.3% yeast extract, 0.3% malt extract, 0.5% peptone, 1% glucose, and 2% agar). The mixture was supplemented with 0.01% chloramphenicol to limit bacterial growth. Plates were incubated at 20 °C and monitored daily to assess colony formation. Selected colonies were streaked onto YM agar plates for subsequent purification. Following purification, strains were suspended in YM broth supplemented with 20% (v/v) glycerol and stored at −80 °C for subsequent use. Cultures of the obtained isolates were preserved in the Microbiology Lab at Nanyang Normal University, Henan, China. All collected isolates and their origins are presented in Table 1.

**Table 1.** Yeast strains and origins investigated in this study.

Strain	Source	Location
*Kockovaellaiteae*		
NYNU 239240^T^	Leaf of *Iteayunnanensis*	East Mountain Park, Guiyang City, Guizhou Province, China (26°45'26"N, 106°21'31"E)
NYNU 239246	Leaf of *Iteayunnanensis*	East Mountain Park, Guiyang City, Guizhou Province, China (26°45'26"N, 106°21'31"E)
*Kockovaellaquanzhouensis*		
NYNU 224192^T^	Leaf of *Ilexasprella*	Qingyuan Mountain, Quanzhou City, Fujian Province, China (25°7'41"N, 118°44'7"E)
NYNU 22425	Leaf of *Myrica* sp.	Qingyuan Mountain, Quanzhou City, Fujian Province, China (25°7'41"N, 118°44'7"E)
*Kockovaellasambucuse*		
NYNU 22942^T^	Leaf of *Sambucuschinensis*	Guiyang Botanical Garden, Guiyang City, Guizhou Province, China (26°34'51"N, 106°42'36"E)

### ﻿Phenotypic characterization

Morphological, physiological, and biochemical characters were examined according to the standard methods described by Kurtzman et al. (2011). To induce sexual state, single or paired strains were mixed on corn meal agar (CMA; 2% cornmeal infusion and 2% agar), potato dextrose agar (PDA; 20% potato infusion, 2% glucose, and 1.5% agar), and V8 agar (10% V8 juice and 2% agar). The plates were then incubated at 20 °C for up to 8 weeks (Li et al. 2020). Ballistoconidium formation was tested using the inverted-plate method (do Carmo-Sousa and Phaff 1962) after two weeks of incubation on CMA at 17 °C. Glucose fermentation was tested in a liquid medium using Durham fermentation tubes. Carbon and nitrogen assimilation capacities were examined in a liquid medium, with nitrogen tests using a starved inoculum (Kurtzman et al. 2011). Growth at various temperatures (15, 20, 25, 30, 35, and 37 °C) was evaluated through cultivation on YM agar plates. Cell morphology was examined with a LEICA DM2500 camera (LECIA, Wetzlar, Germany) and LASV4.13 software. All new taxonomic descriptions and proposed names were submitted to the MycoBank database (http://www.mycobank.org; 17 June 2024).

### ﻿DNA extraction, PCR amplification, and sequencing

Genomic DNA was extracted from each strain using the Ezup Column Yeast Genomic DNA Purification Kit, according to the manufacturer’s directions (Sangon Biotech Co., Shanghai, China). The ITS region, D1/D2 domain of the LSU rRNA, and a partial segment RPB1 were amplified using the primers ITS1/ITS4 (White et al. 1990), NL1/NL4 (Kurtzman and Robnett 1998), and RPB1-Af and RPB1-Cr (Kurtzman and Robnett 2003), respectively. Amplifications were performed in a 25 µL reaction volume consisting of 9.5 µL of ddH_2_O, 12.5 µL of Taq 2X PCR Master Mix with blue dye (Sangon Biotech Co., Shanghai, China), 1 µL of DNA template, and 1 µL of each primer. The ITS region and D1/D2 domain were amplified using an initial denaturation step of 2 min at 95 °C, followed by 35 cycles of 30 s at 95 °C, 30 s at 51 °C, 40 s at 72 °C, and a final extension of 10 min at 72 °C (Toome et al. 2013). Amplification of the partial RPB1 gene was performed using a touchdown PCR protocol as described by Wang et al. (2014). PCR products were then purified and sequenced by Sangon Biotech Co., Ltd (Shanghai, China) using the same primers. The identity and accuracy of each sequence were determined by comparing them to sequences in GenBank. Assembly was performed with BioEdit v.7.1.3.0 (Hall 1999). All newly generated sequences were deposited in the GenBank database (https://www.ncbi.nlm.nih.gov/genbank/), and the accession numbers are presented in Table 2.

**Table 2.** Species name, strain numbers, and GenBank accession numbers included in phylogenetic analyses. Entries in bold represent newly generated sequences. The superscript ^T^ indicates type strain.

Taxa name	Strain number	Locality	GenBank accession numbers		
			ITS	LSU D1/D2	RPB1
*Fellomycesborneensis*	CBS 8282^T^	Indonesia	NR_073336	NG_057663	KF036458
*Fellomycespenicillatus*	CBS 5492^T^	Germany	NR_073217	NG_070551	KF036464
*Fellomycespolyborus*	CBS 6072^T^	South Africa	NR_073238	NG_057660	KF036465
*Fellomyceshorovitziae*	CBS 7515^T^	Germany	NR_073234	NG_057659	KF036461
*Kockovaellabarringtoniae*	CBS 9811^T^	Thailand	KY103846	NG_058315	KF036487
*Kockovaellacalophylli*	CBS 8962^T^	Vietnam	NR_155238	NG_070554	KF036488
*Kockovaellachinensis*	CBS 8278^T^	China	NR_073258	NG_069410	KF036459
*Kockovaellacucphuongensis*	JCM 10840^T^	Vietnam	NR_155210	NG_068957	KF036489
*Kockovaelladistylii*	CBS 8545^T^	Japan	NR_077101	NG_057680	–
*Kockovaellafuzhouensis*	CBS 8243^T^	China	AF444484	NG_058316	KF036460
*Kockovaellahaikouensis*	CGMCC 2.3443^T^	China	NR_174724	MK050274	MK849163
*Kockovaellaimperatae*	CBS 7554^T^	Thailand	NR_077104	AF189862	KF036490
***Kockovaellaiteae***	**NYNU 239240^T^**	**China**	**OR958773**	**OR958772**	**PP755337**
***Kockovaellaiteae***	**NYNU 239246**	**China**	**PP752297**	**PP752296**	**PP755338**
*Kockovaellaischaemi*	CGMCC 2.3565^T^	China	NR_174725	MK050276	MK849182
*Kockovaellalibkindii*	CBS 12685^T^	Brazil	JQ861271	JQ861271	–
*Kockovaellalichenicola*	CBS 8315^T^	China	NR_073338	NG_069411	KF036462
*Kockovaellalitseae*	JCM 10838^T^	Vietnam	NR_155209	NG_068956	KF036491
*Kockovaellamachilophila*	CBS 8607^T^	Japan	NR_077099	NG_057681	KF036492
*Kockovaellamexicana*	CBS 8279^T^	Mexico	NR_164408	KY108124	KF036463
*Kockovaellanitrophila*	CGMCC 2.3465^T^	China	NR_174726	MK050278	MK050278
*Kockovaellaogasawarensis*	CBS 8544^T^	Japan	NR_073264	NG_057679	–
*Kockovaellaphaffii*	CBS 8608^T^	Japan	NR_077098	NG_058317	KF036493
*Kockovaellaprillingeri*	CBS 8308^T^	Thailand	NR_073337	KY108126	KY108126
***Kockovaellaquanzhouensis***	**NYNU 224192^T^**	**China**	**OP278691**	**OP278690**	**PP755336**
***Kockovaellaquanzhouensis***	**NYNU 22425**	**China**	**PP752295**	**PP752294**	**PP755335**
*Kockovaellasacchari*	CBS 8624^T^	Thailand	NR_077102	NG_058318	**KF036494**
***Kockovaellasambucuse***	**NYNU 22942^T^**	**China**	**OP566879**	**OP566878**	–
*Kockovaellaschimae*	CBS 8610^T^	Japan	NR_137140	NG_058319	KF036495
*Kockovaellasichuanensis*	CBS 8318^T^	China	NR_073259	AF189879	KF036466
*Kockovaellathailandica*	CBS 7552^T^	Thailand	NR_077103	NG_057650	KF036496
*Kockovaellavietnamensis*	JCM 10841^T^	Vietnam	NR_077111	NG_058320	KF036497
*Sterigmatosporidiumpolymorphum*	CBS 8088^T^	Germany	NR_111071	AF075480	KF036418

### ﻿Phylogenetic analysis

In addition to the newly generated sequences, additional related sequences were also downloaded from GenBank (Table 2) for phylogenetic analyses. The combined ITS, LSU, and RPB1 sequence dataset was used to explore the phylogenetic positions of the newly isolated strains within *Kockovaella*. All *Kockovaella* and *Fellomyces* species listed in Table 2, with available ITS, LSU, and RPB1 sequences, were included as ingroup taxa. *Sterigmatosporidiumpolymorphum* CBS 8088 was used as the outgroup (Gomes et al. 2016). Because previous phylogenetic studies focusing on *Kockovaella* were mainly based on the ITS and LSU regions, a combined ITS and LSU sequences dataset, comprising all species of *Kockovaella* and *Fellomyces* in Table 2, was used to further differentiate species identities within this genus.

Individual locus sequences were aligned using MAFFT v.7.110 (Katoh and Standley 2013) under the G-INI-I option. Poorly aligned regions were excluded and adjusted manually using MEGA v.11 (Tamura et al. 2021). Aligned sequences of the different loci were concatenated with Phylosuit v.1.2.2 (Zhang et al. 2020). Alignments were improved through manual gap adjustments. Ambiguous areas were excluded from the analysis using Aliview (Larsson 2014).

Phylogenetic analyses were conducted using Maximum Likelihood (ML) and Bayesian Inference (BI) methods. The ML method was performed using RAxML v.8.2.3 (Stamatakis 2014) under a GTRGAMMA model with one thousand rapid bootstrap (BS) replicates. For the BI approach, ModelFinder (Kalyaanamoorthy et al. 2017) was used to infer the appropriate substitution model that would best fit the model of DNA evolution for all combined datasets. The BI method was conducted using MrBayes v.3.2.7a (Ronquist et al. 2012) via the CIPRES Science Gateway. Six simultaneous Markov chains were run for 50 million generations, with trees sampled every 1,000 th generation. The first 25% of trees were discarded as burn-in. The remaining trees were used to calculate the Bayesian posterior probabilities (BPPs) for each clade. The resulting trees were visualized with FigTree v.1.4.3 (Andrew 2016). Branches showing BS values ≥ 50% and BPPs ≥ 0.95 indicated at the nodes.

## ﻿Results

### ﻿Molecular phylogeny

**Figure 1.**
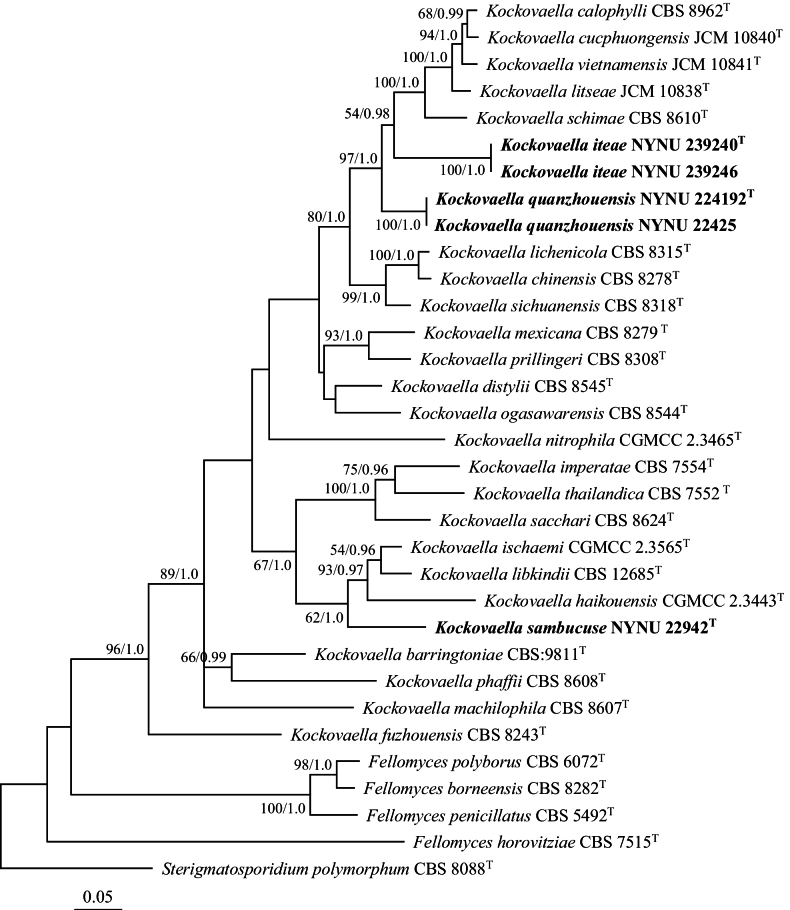
Maximum likelihood phylogenetic tree of *Kockovaella* generated from combined ITS, LSU, and RPB1 sequence data. The tree is rooted with *Sterigmatosporidiumpolymorphum* CBS 8088. Bootstrap values (BS ≥ 50% and BPPs ≥ 0.95) are displayed near branches. Type strain sequences are marked with (T). New species are highlighted in bold.

The combined dataset of ITS, LSU, and RPB1 resulted in an alignment of 1930 characters (ITS: 1–496, LSU: 497–1118, RPB1: 1119–1930). Among them, there were 1120 constant, 155 variable but parsimony non-informative, and 655 parsimony informative characters. ModelFinder recommended the GTR+I+G evolution model for Bayesian inference. Both ML and BI methods produced similar topologies in the main lineages. The ML-derived topology, along with BS values and BPPs above 50% and 0.95, respectively, is presented (Fig. 1). The phylogeny confirmed *Kockovaella* as a distinct genus (BS/92%; BPP/1). The five newly isolated strains formed three distinct and well-supported groups, separate from other *Kockovaella* species.

**Figure 2.**
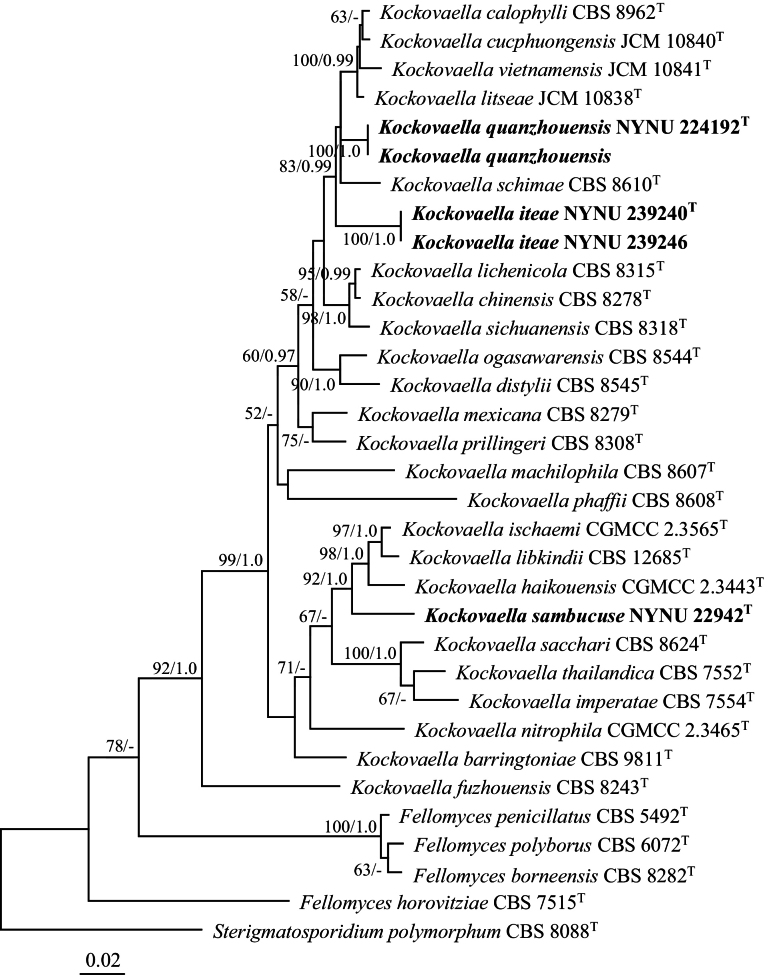
Maximum likelihood phylogenetic tree of *Kockovaella* generated from combined ITS and LSU sequence data. The tree is rooted with *Sterigmatosporidiumpolymorphum* CBS 8088. Bootstrap values (BS ≥ 50% and BPPs ≥ 0.95) are displayed near branches. Type strain sequences are marked with (T). New species are highlighted in bold.

The combined dataset of ITS and LSU sequences produced a concatenated alignment of 1,118 characters, including 817 constant, 88 variable but parsimony non-informative, and 213 parsimony informative characters. The GTR+I+G evolution model was also adopted for this dataset in Bayesian inference. The ML and BI methods yielded similar topologies in the main lineages. The ML-derived topology, with BS values and BPPs above 50% and 0.95, respectively, is shown (Fig. 2). This tree revealed 23 known *Kockovaella* species, while the newly isolated strains formed three independent groups, consistent with the combined ITS, LSU, and RPB1 dataset phylogeny.

Groups NYNU 224192 and NYNU 239240, each containing two strains, clustered with *K.calophylli*, *K.cucphuongensis*, *K.litseae*, *K.schimae*, and *K.vietnamensis* in all combined dataset trees (Figs 1, 2). Strains in the NYNU 224192 group had identical ITS and D1/D2 sequences, indicating that they are conspecific. Strains in the NYNU 239240 group, also with identical ITS and D1/D2 sequences, differed from the NYNU 224192 group by 8 nucleotide (nt) (~1.3%) substitutions and 29 nt (~5.8%) mismatches in the D1/D2 and ITS regions, respectively. These two groups differed from their five closest known species by 4–9 nucleotide (nt) (~0.7–1.5%) substitutions and 14–13 nt (~2.8–4.4%) mismatches in the D1/D2 and ITS regions, respectively. Strain NYNU 22942 clustered with *K.haikouensis*, *K.ischaemi*, and *K.libkindii* with 62% BS and 1.0 BPPs support in the combined ITS, LSU, and RPB1 phylogenetic tree (Fig. 1). It formed a well-supported clade with these species in the combined ITS and LSU dataset tree (92% BS, 1.0 BPPs; Fig. 2), differing from its nearest relatives by 9–10 nucleotide (nt) (~1.5–1.7%) substitutions and 25–27 nt (~4.7–5.1%) mismatches in the D1/D2 and ITS regions, respectively.

The above sequence comparisons suggested that the five novel strains represent three novel species within the genus *Kockovaella*.

### ﻿Taxonomy

#### ﻿ Kockovaella iteae

Taxon classificationFungiTremellalesCuniculitremaceae

C.Y. Chai & F.L. Hui sp. nov.

E3FFD6CA-9BE8-53B3-84E4-F80879939837

854381

Fig. 3

##### Etymology.

The specific epithet *iteae* refers to *Itea*, the plant genus from which the type strain was isolated.

##### Type.

China • Guizhou Prov.: Guiyang City, East Mountain Park, in the phylloplane of *Iteayunnanensis*, 15 Sept 2023, D. Lu, NYNU 239240 (holotype GDMCC 2.503^T^ preserved as a metabolically inactive state, culture ex-type PYCC 9996).

##### Description.

On YM agar after 7 days at 20 °C, the streak culture is white to cream-colored, butyrous, smooth and glistening, with an entire margin. After 7 days in YM broth at 20 °C, cells are ellipsoidal or ovoid, 1.5–3.6 × 3.6–5.5 μm, single or pairs, and reproduced by polar budding and the formation of stalked conidia. The conidia are separated at the distal end of the stalks from parent cells. After 1 month at 20 °C, a ring and sediment are present. In Dalmau plate culture on CMA, pseudohyphae are not formed. Sexual structures are not observed on PDA, CMA or V8 agar. Ballistoconidia are symmetrical and apiculate, 1.8–2.4 × 2.7–3.3 μm. Glucose fermentation is absent. Glucose, inulin (delayed and weak), sucrose, raffinose, melibiose, galactose, lactose, trehalose, maltose, melezitose, cellobiose, salicin (delayed and weak), L-rhamnose, D-xylose, L-arabinose, D-arabinose (delayed), 5-keto-D-gluconate (delayed and weak), D-ribose (delayed), erythritol (delayed), ribitol, galactitol, D-mannitol, D-glucitol, myo-inositol, succinate, citrate, D-glucosamine, N-acetyl-D-glucosamine, 2-keto-D-gluconate (delayed), D-glucuronate, and glucono-1.5-lactone are assimilated as sole carbon sources. Methyl-α-D-glucoside, L-sorbose, methanol, ethanol, glycerol, DL-lactate, and D-gluconate are not assimilated. Ethylamine (delayed) and L-lysine are assimilated as sole nitrogen sources. Nitrate, nitrite, and cadaverine are not assimilated. Maximum growth temperature is 25 °C. Growth in vitamin-free medium is positive. Growth on 50% (w/w) glucose-yeast extract agar is negative. Starch-like substances are not produced. Urease activity is positive. Diazonium Blue B reaction is positive.

**Figure 3.**
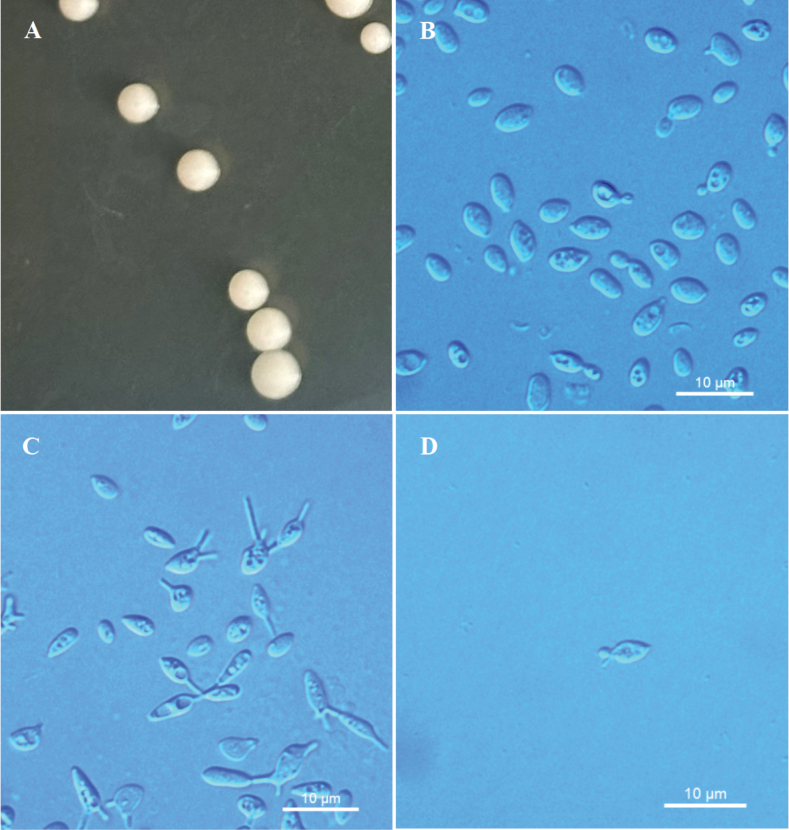
Morphological characteristics of *Kockovaellaiteae* sp. nov. NYNU 239240^T^**A** colony morphology on YM agar after growth for 7 d at 20 °C **B** budding cells after growth for 7 d in YM broth at 20 °C **C** stalked conidia on PDA after growth for 7 d at 20 °C **D** ballistoconidia on CM agar after growth for 7 d at 20 °C. Scale bars: 10 μm.

##### Additional strain examined.

China • Guizhou Prov.: Guiyang City, East Mountain Park, in the phylloplane of *Iteayunnanensis*, 15 Sept 2023, D. Lu, NYNU 239246.

##### GenBank accession numbers.

holotype GDMCC 2.503^T^ (ITS: OR958773, D1/D2: OR958772, RPB1: PP755337); additional strains NYNU 239246 (ITS: PP752297, D1/D2: PP752296, RPB1: PP755338).

##### Note.

Physiologically, *Kockovaellaiteae* sp. nov. differs from six closely related species, *K.calophylli*, *K.cucphuongensis*, *K.litseae*, *K.quanzhouensis*, *K.schimae*, and *K.vietnamensis*, in its ability to assimilate inulin and ethylamine (Table 3).

**Table 3.** Physiological and biochemical characteristics differing between the new species and closely related species.

Characteristics	1	2*	3*	4*	5*	6*	7	8	9*	10*	11*
Carbon assimilation											
Inulin	d/w	–	–	–	–	–	–	–	–	+	–
L-Sorbose	–	–	d/w	W	w	d/w	d/w	d	–	–	–
D-Arabinose	d	d	w	D	d	d	–	+	-	w	+
Galactitol	+	+	d/w	D	d	w	–	+	+	+	+
Succinate	+	d/w	d	D	+	+	–	+	v	w	n
Citrate	+	d/w	d	D	w	w	–	+	–	v	–
Glucono-δ-lactone	+	d/w	d/w	D	w	w	–	+	n	n	n
Nitrogen assimilation											
Ethylamine	d	–	–	–	–	–	–	–	d	–	n
Cadaverine	–	–	–	–	–	–	–	–	+	+	n
Growth tests											
Growth at 30 °C	–	–	+	–	–	+	+	+	+	+	**n**

Species: 1, *K.iteae*; 2, *K.schimae*; 3, *K.calophylli*; 4, *K.cucphuongensis*; 5, *K.litseae*; 6, *K.vietnamensis*; 7, *K.quanzhouensis*; 8, *K.sambucuse*; 9, *K.haikouensis*; 10, *K.ischaemi*; 11, *K.libkindii.* +, positive reaction; –, negative reaction; d, delayed positive; w, weakly positive; n, data not available. All data from this study, except those marked with *, which were obtained from the original description (Lopandic et al. 2011; Takashima and Nakase 2011; Gomes et al. 2016).

#### ﻿ Kockovaella quanzhouensis

Taxon classificationFungiTremellalesCuniculitremaceae

C.Y. Chai & F.L. Hui sp. nov.

F369DEFA-9875-5342-B163-5D00AEFD0791

854382

Fig. 4

##### Etymology.

The specific epithet *qingyuanensis* refers to the geographic origin of the type strain, Qingyuan Mountain, Quanzhou, Fujian.

##### Type.

China • Fujian Prov.: Quanzhou City, Qingyuan Mountain, in the phylloplane of *Ilexasprella*, 12 Mar 2022, W.T. Hu & S.B. Chu, NYNU 224192 (holotype GDMCC 2.325^T^ preserved as a metabolically inactive state, culture ex-type PYCC 9950).

##### Description.

On YM agar after 7 days at 20 °C, the streak culture is cream to pale yellow, butyrous, smooth and glistening, with an entire margin. After 7 days in YM broth at 20 °C, cells are ovoid, 2.1–4.9 × 3.3–5.6 μm, single or pairs, and reproduced by polar budding and the formation of stalked conidia. The conidia are separated at the distal end of the stalks from parent cells. After 1 month at 20 °C, a ring and sediment are present. In Dalmau plate culture on CMA, pseudohyphae and hyphae are not formed. Sexual structures are not observed on PDA, CMA or V8 agar. Ballistoconidia are symmetrical and apiculate, 3.7–4.2 × 7.9–8.0 μm. Glucose fermentation is absent. Glucose, sucrose, raffinose, melibiose, galactose, lactose, trehalose, maltose, melezitose, cellobiose, L-sorbose (delayed and weak), L-rhamnose, D-xylose, L-arabinose, D-ribose, D-mannitol, D-glucitol, D-gluconate (delayed), D-glucosamine, N-acetyl-D-glucosamine, and D-glucuronate are assimilated as sole carbon sources. Inulin, methyl-α-D-glucoside, salicin, D-arabinose, 5-keto-D-gluconate, methanol, ethanol, glycerol, erythritol, ribitol, galactitol, myo-inositol, DL-lactate, succinate, citrate, 2-keto-D-gluconate, and glucono-1.5-lactone are not assimilated. L-Lysine is assimilated as sole nitrogen sources. Nitrate, nitrite, ethylamine, and cadaverine are not assimilated. Maximum growth temperature is 30 °C. Growth in vitamin-free medium is positive. Growth on 50% (w/w) glucose-yeast extract agar is negative. Starch-like substances are not produced. Urease activity is positive. Diazonium Blue B reaction is positive.

**Figure 4.**
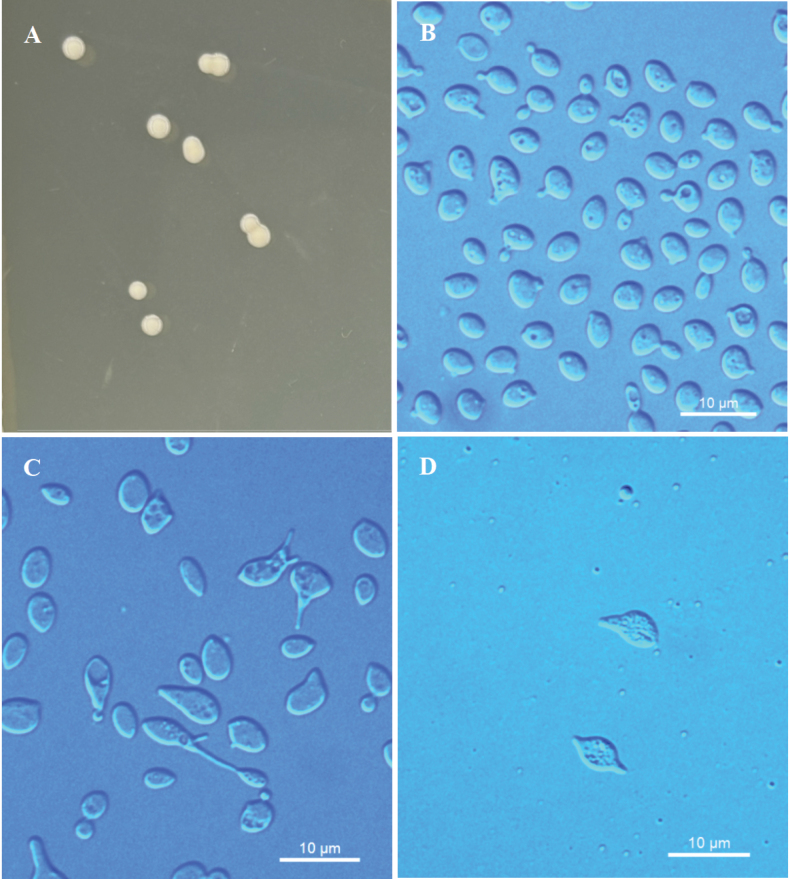
Morphological characteristics of *Kockovaellaquanzhouensis* sp. nov. NYNU 224192^T^**A** colony morphology on YM agar after growth for 7 d at 20 °C **B** budding cells after growth for 7 d in YM broth at 20 °C **C** stalked conidia on PDA after growth for 7 d at 20 °C **D** ballistoconidia on CM agar after growth for 7 d at 20 °C. Scale bars: 10 μm.

##### Additional strain examined.

China • Fujian Prov.: Quanzhou City, Qingyuan Mountain, in the phylloplane of *Myrica* sp., 12 Mar 2022, W.T. Hu & S.B. Chu, NYNU 22425.

##### GenBank accession numbers.

holotype GDMCC 2.325^T^ (ITS: OP278691, D1/D2: OP278690, RPB1: PP755336); additional strains NYNU 22425 (ITS: PP752295, D1/D2: PP752294, RPB1: PP755335).

##### Note.

Physiologically, *Kockovaellaquanzhouensis* sp. nov. differs from six closely related species, *K.calophylli*, *K.cucphuongensis*, *K.litseae*, *K.iteae*, *K.schimae*, and *K.vietnamensis*, in its inability to assimilate D-arabinose, galactitol, succinate, citrate and glucono-1.5-lactone (Table 3).

#### ﻿ Kockovaella sambucuse

Taxon classificationFungiTremellalesCuniculitremaceae

C.Y. Chai & F.L. Hui sp. nov.

E63BEB1A-637A-54BD-957A-201523F4EE41

854383

Fig. 5

##### Etymology.

The specific epithet *sambucuse* refers to *Sambucus*, the plant genus from which the type strain was isolated.

##### Type.

China • Guizhou Prov.: Guiyang City, Guiyang Botanical Garden, in the phylloplane of *Sambucuschinensis*, Aug 2022, L. Zhang and F.L. Hui, NYNU 22942 (holotype GDMCC 2.313^T^ preserved as a metabolically inactive state, culture ex-type PYCC 9951).

##### Description.

On YM agar after 7 days at 20 °C, the streak culture is white to cream-colored, butyrous, smooth and glistening, with an entire margin. After 7 days in YM broth at 20 °C, cells are ovoid, 2.1–3.3 × 3.3–4.7 μm, and single or pairs, budding is polar. After 1 month at 20 °C, a ring and sediment are present. In Dalmau plate culture on CMA, pseudohyphae and hyphae are not formed. Sexual structures are not observed on PDA, CMA or V8 agar. Ballistoconidia are ellipsoidal or somewhat kidney-shaped, 3.4–4.9 × 5.2–6.8 μm. Glucose fermentation is absent. Glucose, sucrose, raffinose, melibiose, galactose, lactose, trehalose, maltose, melezitose, cellobiose, salicin, L-sorbose (delayed), L-rhamnose (delayed), D-xylose, L-arabinose, D-arabinose, D-ribose, glycerol (delayed), ribitol, galactitol, D-mannitol, D-glucitol, DL-lactate (delayed and weak), succinate, citrate, D-glucosamine, N-acetyl-D-glucosamine, 2-keto-D-gluconate (weak), D-glucuronate and glucono-1.5-lactone are assimilated as sole carbon sources. Inulin, methyl-α-D-glucoside, 5-keto-D-gluconate, methanol, ethanol, erythritol, myo-inositol, and D-gluconate are not assimilated. L-Lysine is assimilated as sole nitrogen sources. Nitrate, nitrite, ethylamine and cadaverine are not assimilated. Maximum growth temperature is 30 °C. Growth in vitamin-free medium is positive. Growth on 50% (w/w) glucose-yeast extract agar is negative. Starch-like substances are not produced. Urease activity is positive. Diazonium Blue B reaction is positive.

**Figure 5.**
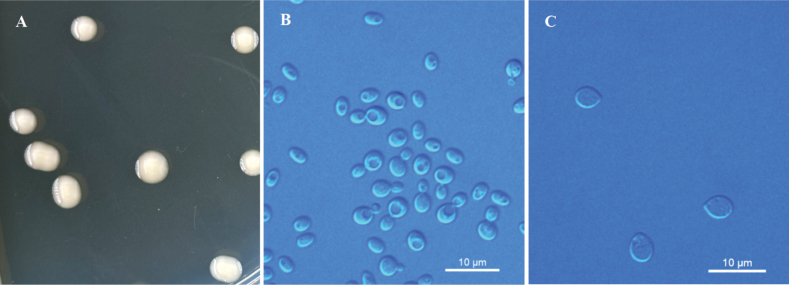
Morphological characteristics of *Kockovaellasambucuse* sp. nov. NYNU 22942^T^**A** colony morphology on YM agar after growth for 7 d at 20 °C **B** budding cells after growth for 7 d in YM broth at 20 °C **C** ballistoconidia on CM agar after growth for 7 d at 20 °C. Scale bars: 10 μm.

##### GenBank accession numbers.

holotype GDMCC 2.313^T^ (ITS: OP566879, D1/D2: OP566878).

##### Note.

Physiologically, *Kockovaellasambucuse* sp. nov. differs from three closely related species, *K.haikouensis*, *K.ischaemi*, and *K.libkindii*, in its ability to assimilate L-sorbose and its inability to assimilate cadaverine (Table 3).

## ﻿Discussion

Phylogenetic analyses grouped 26 species of *Kockovaella* together (Figs 1, 2), including three new species from China: *K.iteae* sp. nov., *K.quanzhouensis* sp. nov., and *K.sambucuse* sp. nov. Our results are consistent with previous observations (Gomes et al. 2016; Li et al. 2020), and provide additional insights into the phylogeny and taxonomy of *Kockovaella*.

*Kockovaellasambucuse* sp. nov. described in this study was represented by only one strain from our isolations. Despite a number of samples collected in different locations for two consecutive years, we were unable to confirm the occurrence of this yeast to obtain additional strains. The description of single-strain species will add to an understanding of yeast phylogeny and species diversity, which would be unknown if new species descriptions were limited to those taxa for which multiple strains were available (Kurtzman 2010).

*Fellomyceshorovitziae* was first reported in Germany by Spaaij et al. (1991) based on phenotypic characteristics. However, our phylogenetic analysis did not support its placement in *Fellomyces*, despite its morphological similarity to other species in the genus, as it forms conidia on stalks (Lopandic et al. 2011). This result is similar to the results of previous phylogenetic analyses based on the single LSU sequence and the combined ITS and LSU sequences (Gomes et al. 2016; Li et al. 2020). Therefore, further analyses using more molecular or genomic data are needed to clarify its phylogenetic position.

*Kockovaella* species are widely distributed across various habitats. They are commonly identified as epiphytic fungi on flowers (Yue 1982), leaves (Canete-Gibas et al. 1998; Hamamoto et al. 1998; Takashima and Nakase 1998; Luong et al. 2000; Fungsin et al. 2002; Lopandic et al. 2011; Li et al. 2020), and lichens (Prillinger et al. 1997; Lopandic et al. 2005b) in temperate and subtropical climate regions. *K.libkindii*, for example, has been found in water cavities (Gomes et al. 2016), where it forms a minor component of the yeast community, likely vectored by insects visiting these microhabitats (Gomes et al. 2016). In this study, three new *Kockovaella* species were associated with plant leaves, similar to other species in the genus. Other species identified from these samples include *Bulleraalba*, *Derxomycesboekhoutii*, *Erythrobasidiumprimogenitum*, *Moesziomycesantarcticus*, *Moesziomycesaphidis*, *Symmetrosporacoprosmae*, and *Tilletiopsiswashingtonensis*, all common representatives in the phyllosphere (Nakase 2000; Kruse et al. 2017). The discovery of these three new species highlights the widespread natural distribution of *Kockovaella* species on plants, emphasizing the need for extensive sampling and detailed molecular and phenotypic analyses to fully understand their global diversity.

## ﻿Conclusions

In the present study, five phyllosphere-inhabiting yeast strains were identified as three new *Kockovaella* species, *K.iteae* sp. nov., *K.quanzhouensis* sp. nov., and *K.sambucuse* sp. nov., based on morphological and molecular phylogenetic analyses, which provides us with further understanding of this genus diversity in China. In the future, we firmly believe that more and more species of the genus will be isolated from more plants around the world.

## Supplementary Material

XML Treatment for Kockovaella iteae

XML Treatment for Kockovaella quanzhouensis

XML Treatment for Kockovaella sambucuse

## ﻿Additional information

### Author ORCIDs

Chun-Yue Chai https://orcid.org/0000-0003-0284-5560

Qiu-Hong Niu https://orcid.org/0000-0003-1695-7117

Feng-Li Hui https://orcid.org/0000-0001-7928-3055

### Data availability

The datasets presented in this study can be found in online repositories. The names of the reposi-tory/repositories and accession number(s) can be found in the article.
